# Bioinformatics Analysis of the Inflammation-Associated lncRNA-mRNA Coexpression Network in Type 2 Diabetes

**DOI:** 10.1155/2023/6072438

**Published:** 2023-02-22

**Authors:** Linjuan Huang, Shengxi Xiong, Hanshuang Liu, Min Li, Ranran Zhang, Yan Liu, Xiaolei Hu

**Affiliations:** The Department of Endocrinology, The First Affiliated Hospital of Bengbu Medical College, Bengbu 233000, China

## Abstract

**Introduction:**

Diabetes is a chronic inflammatory state, and a key role of lncRNAs in diabetes complications is a new area of research.

**Methods:**

In this study, key lncRNAs related to diabetes inflammation were identified by RNA-chip mining and lncRNA-mRNA coexpression network construction and finally verified by RT-qPCR.

**Results:**

We ultimately obtained 12 genes, including A1BG-AS1, AC084125.4, RAMP2-AS1, FTX, DBH-AS1, LOXL1-AS1, LINC00893, LINC00894, PVT1, RUSC1-AS1, HCG25, and ATP1B3-AS1. RT-qPCR assays verified that LOXL1-AS1, A1BG-AS1, FTX, PVT1, and HCG25 were upregulated in the HG+LPS-induced THP-1 cells, and LINC00893, LINC00894, RUSC1-AS1, DBH-AS1, and RAMP2-AS1 were downregulated in the HG+LPS-induced THP-1 cells.

**Conclusions:**

lncRNAs and mRNAs are extensively linked and form a coexpression network, and lncRNAs may influence the development of type 2 diabetes by regulating the corresponding mRNAs. The ten key genes obtained may become biomarkers of inflammation in type 2 diabetes in the future.

## 1. Introduction

Diabetes mellitus (DM) is a chronic metabolic disease that affects approximately 460 million adults aged 20-79 years and accounts for 10% of global health expenditures [1]. Type 2 diabetes mellitus (T2DM) is caused by insulin resistance and/or insulin deficiency and accounts for almost 95% of all diabetes cases [2]. There are multiple environmental and genetic risk factors for T2DM, and significant heterogeneity is observed in these patients [3]. A better understanding of the biological pathways leading to the pathogenesis of type 2 diabetes and its complications may improve patient management. Therefore, the discovery of new screening markers and therapeutic targets is the current focus of type 2 diabetes research.

Diabetes is considered a chronic inflammatory disease, and diabetic inflammation occurs widely in the complications associated with diabetes [4]. Complications of diabetes are a major health and economic burden [5]. Therefore, the central role of inflammation has received extensive attention [6]. Long-stranded noncoding RNAs (lncRNAs) are a hot topic in the study of molecular mechanisms of disease. lncRNAs play a potential role in the development and regression of diabetes [7, 8]. Currently known functions are mostly related to microRNAs (miRNAs), and lncRNAs act as negative regulators of miRNAs to block miRNA binding to target mRNAs and act as competitive endogenous RNAs (ceRNAs) for miRNAs [9]. There are few lncRNA-mRNA coexpression-related studies. In the present study, we performed Pearson's correlation analysis, cluster analysis, KEGG pathway analysis, and GO functional enrichment analysis. We constructed a lncRNA-mRNA coexpression network using Cytoscape to predict key lncRNAs in peripheral blood monocytes of T2DM patients and provide a basis for studying the molecular mechanism of type 2 diabetes occurrence and treatment.

## 2. Materials and Methods

### 2.1. The Aim, Design, and Setting of the Study

RNA microarray technology was used to determine the expression profiles of differential lncRNAs in peripheral blood monocytes of T2DM and normal humans. R language and related bioinformatics tools were used to process the microarray data, perform KEGG pathway and GO function enrichment analyses of differentially expressed lncRNAs, and predict key lncRNA target genes to construct a lncRNA-mRNA coexpression network. The microarray results were validated using RT-qPCR.

### 2.2. The Characteristics of Participants or Description of Materials

Four patients with confirmed T2DM were selected as the T2DM group, and four age- and sex-matched healthy physical examiners were used as normal controls (NC group). Patients with secondary or special types of DM and gestational diabetes mellitus (GDM), patients with acute complications of DM, coinfectious diseases, immune system diseases, and patients with tumors were excluded. The Ethics Committee of the First Affiliated Hospital of Bengbu Medical College reviewed and approved the study, and all study subjects signed an informed consent form. Their peripheral blood monocytes were collected as samples for microarray detection and subsequent validation at the THP-1 cell level. Feature Extraction software (version 12.0.3.1, Agilent Technologies) was used to process the raw images and extract the raw data. Genespring software (version 14.8, Agilent Technologies) was used for quantile normalization and subsequent processing.

### 2.3. Bioinformatics Analysis

The results were obtained after importing the raw data into Genespring software (version 14.8, Agilent) and normalizing the data using the quantile method. The standardized data were filtered, and at least one set of probes marked 100% detected in each group of samples used for comparison was left for subsequent analysis. Differential genes were screened using the *p* value of the *t* test with a *p* value ≤ 0.05. Differentially expressed genes were analyzed by GO and KEGG to determine their biological functions or pathways. Unsupervised hierarchical clustering of differential genes was performed to demonstrate the expression pattern of differential genes between different samples using a heatmap.

### 2.4. Cell Culture and Model Construction

THP-1 cells were obtained from the cell bank of Bengbu Medical College and cultured in 1640 medium (BasalMedia) +10% fetal bovine serum (EXCell Bio) and 1% penicillin and streptomycin (NCM Biotech) at 37°C in a humidified atmosphere of 5% CO_2_. THP-1 cells were induced to become macrophages with 50 ng/mL PMA for 48 h. For subsequent steps, THP-1-induced macrophages were cultured for 2 days using RPMI 1640 medium with glucose concentrations of 5.5 mmol/L for the normal control (NC) and HG+LPS group with glucose (25 mmol/L) + LPS (100 ng/mL) + IFN-*γ* (20 ng/mL) in RPMI 1640 culture medium that were used to culture THP-1-induced macrophages for 2 days.

### 2.5. RNA Sequencing Sample Preparation and Steps

Total RNA was extracted using TRIzol (Invitrogen) reagent and purified using a QIAGEN RNeasy Kit. A total of 250 ng of purified total RNA was used for labeling and amplification. The RNA was first reverse transcribed into the first strand of cDNA using an AffinityScript-RT kit and promoter primer, and the second strand of cDNA was generated using anti-sense promoter. cRNA was generated by adding T7 RNA polymerase and amplifying the second strand of cDNA. Cyanine-3-CTP (Cy3), a fluorescent dye, was used for labeling, and the QIAGEN RNeasy Kit was used for purification after labeling. Hybridization was performed at 65°C for 17 h rolling, and the original images were scanned using an Agilent Scanner G5761A (Agilent Technologies) after elution.

### 2.6. RT-qPCR

Total RNA was extracted and reverse transcribed into cDNA using the Abscript cDNA First-Strand Synthesis Kit reverse transcriptase (Abclonal Technology). lncRNA expression levels were determined using 2× Universal SYBR Green Fast qPCR Mix (Abclonal Technology), and specific primers were used to determine the expression levels of three randomly selected lncRNAs that were differentially expressed in HG+LPS-induced THP-1 cells (Table 1). RT-qPCR was performed in triplicate using the following temperature profile: 95°C predenaturation for 3 minutes, followed by 40 cycles consisting of 95°C for 5 seconds and 60°C for 34 s. GAPDH was used as an internal reference gene. The expression level of each lncRNA was analyzed using the 2^-*ΔΔ*CT^ method, and the expression level of each lncRNA is expressed as a fold change between the control THP-1 group and the HG+LPS-induced THP-1 group.

### 2.7. Statistical Analysis

Statistical analysis was performed using GraphPad Prism 8.0.1. Significance was determined using unpaired t tests for two samples. *p* < 0.05 (^∗^ or ^#^) was considered statistically significant. (^∗^ or ^#^*p* < 0.05; ^∗∗^ or ^##^*p* < 0.01; ^∗∗∗^ or ^###^*p* < 0.001).

## 3. Results

### 3.1. Analysis of RNA-Chip Results

**Figure 1:**
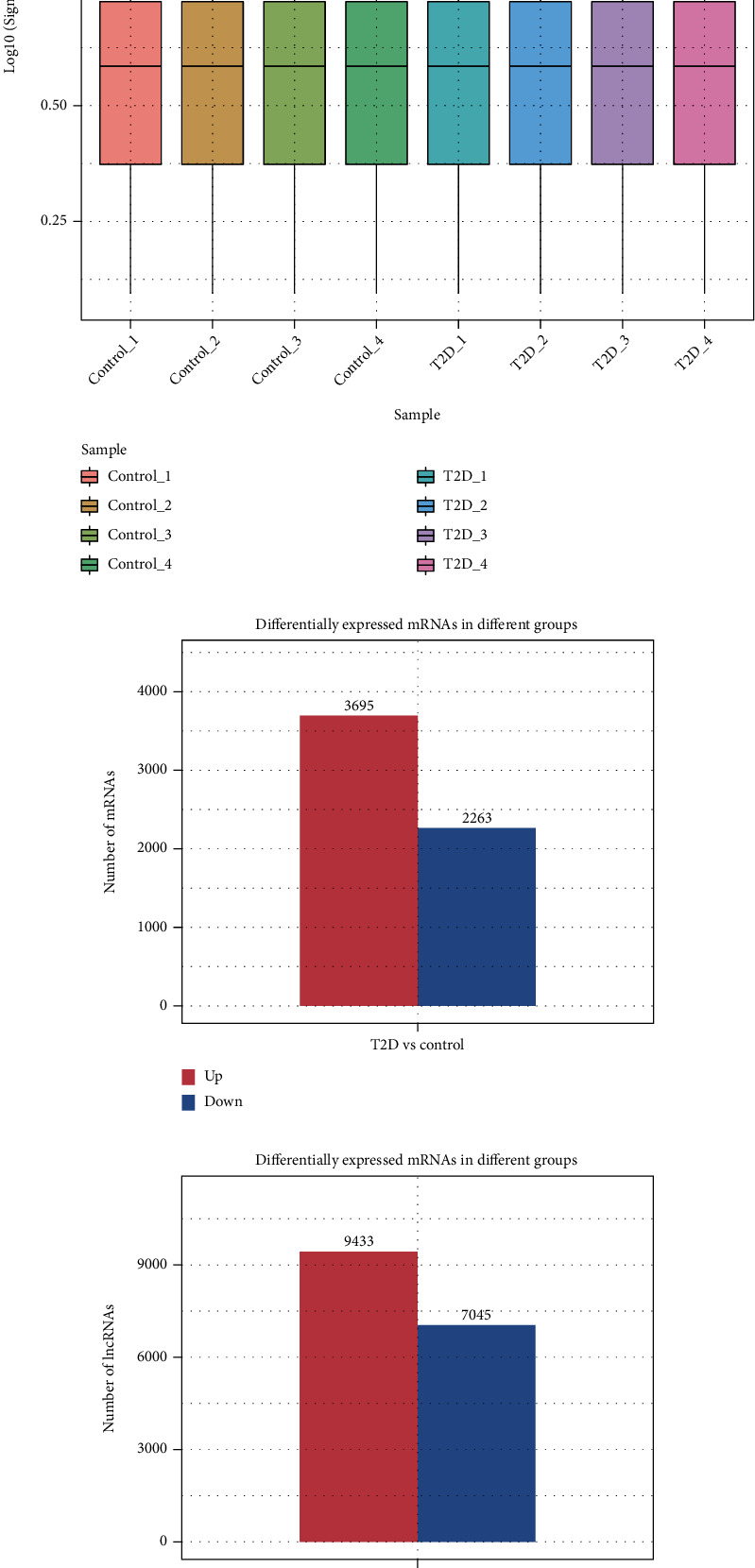
Overview of lncRNAs identified in control healthy individuals and diabetic patients. (a) Bar graph showing the distribution of differentially expressed lncRNAs and mRNAs on human chromosomes. (b) Sample box plot depicting the distribution statistics of RNA expression values. (c) The correlation analysis plot shows the correlation coefficients calculated between the different samples. (d, e) Frequency plots show the frequency distribution of mRNA and lncRNA up- and downregulation in controls and diabetic patients. (f, g) Volcano plot. The red and blue dots indicate dysregulated lncRNAs with >2-fold decreased and increased expression in diabetes mellitus.

**Figure 2:**
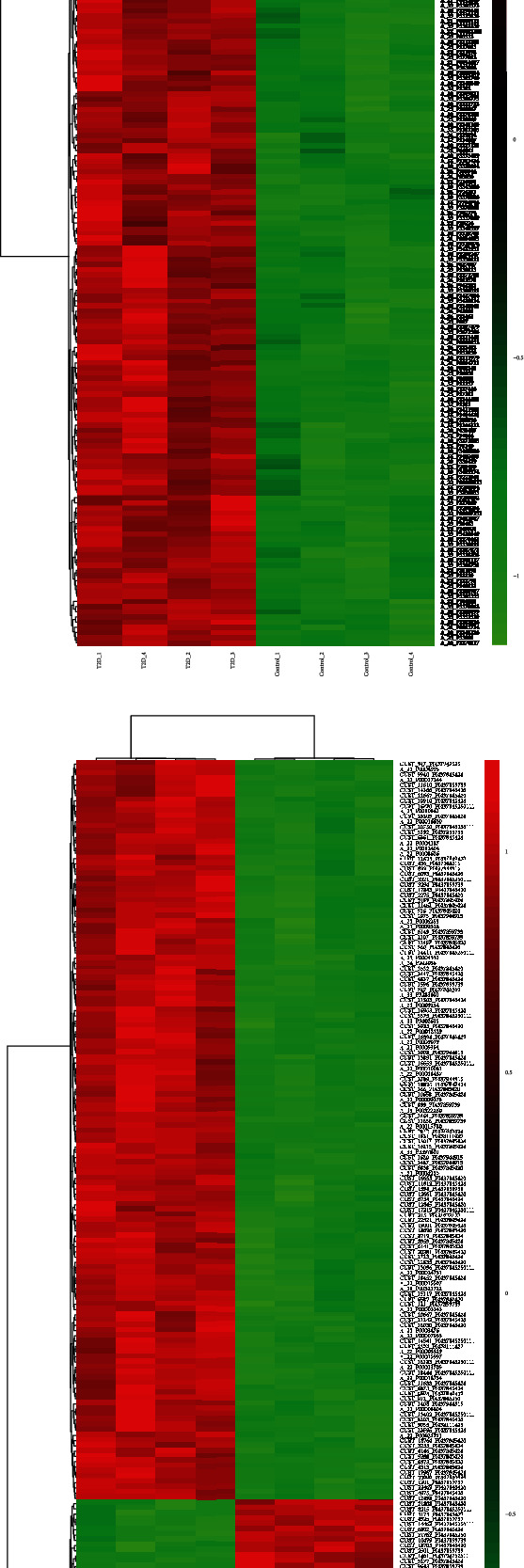
Hierarchical clustering. (a, b) Clustering analysis of differential mRNA and lncRNA expression levels. For the clustering analysis of differentially expressed mRNAs, we selected the top 200 with the smallest *p* value for the clustering graph.

To investigate the expression of lncRNAs in diabetic patients, we performed in-depth RNA-chip experiments on four healthy individuals and four diabetic patients. We identified 16,478 differential lncRNAs from 8 samples and further counted the distribution maps of differentially expressed lncRNAs and mRNAs in human chromosomes (Figure 1(a)). The RNA expression value distribution statistics were further represented by a sample box plot (Figure 1(b)), and correlation analysis plots (Figure 1(c)) were generated. The normalized data were subsequently analyzed, and 16478 differentially expressed lncRNAs were obtained, of which 9433 were upregulated and 7045 were downregulated. There were 5958 differentially expressed mRNAs, of which 3695 were upregulated, and 2263 were downregulated. As shown in Figures 1 and 2, the mRNA volcano plot (Figure 1(f)), lncRNA volcano plot (Figure 1(g)), mRNA frequency plot (Figure 1(d)), lncRNA frequency plot (Figure 1(e)), mRNA hierarchical clustering (Figure 2(a)), and lncRNA hierarchical clustering (Figure 2(b)) indicated that the lncRNA and mRNA expression profiles of diabetic patients and healthy individuals were different.

### 3.2. mRNA Differential Gene GO and KEGG Enrichment Analysis

**Figure 3:**
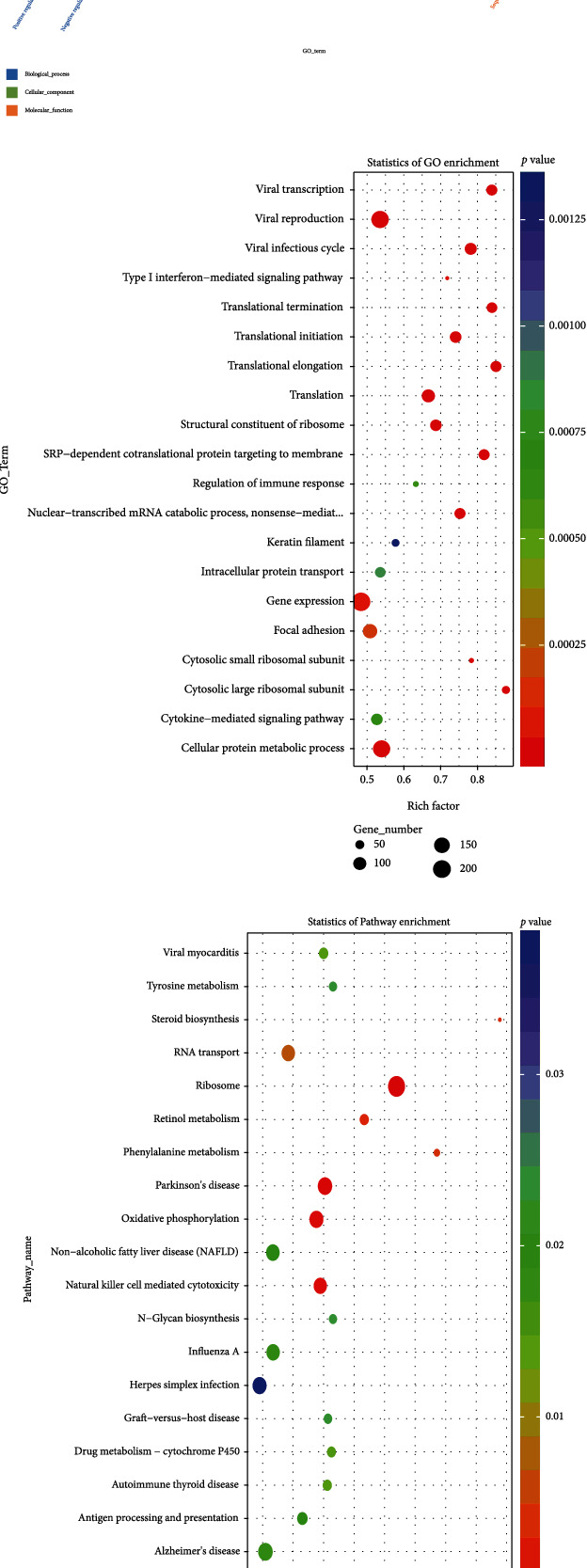
(a) GO enrichment histogram, (b) GO enrichment histogram, and (c) KEGG enrichment scatterplot show the functional profiles of differentially expressed parental mRNA genes in controls and diabetic patients.

To further investigate the functional differences in mRNA genes between diabetic patients and normal subjects, we performed GO and KEGG analyses. The GO enrichment histogram (Figure 3(a)), GO enrichment scatter plot (Figure 3(b)), and KEGG enrichment scatter plot (Figure 3(c)) show the functional profiles of differentially expressed mRNA parental genes in healthy individuals and diabetic patients. The histogram of the GO enrichment analysis results shows that the main biological process is transcription, DNA-dependent, the main cellular component is the nucleus, and the main molecular functional class is protein binding. GO enrichment analysis contains a variety of inflammation-related GO terms including regulation of acute inflammatory response, chronic inflammatory response, negative regulation of inflammatory response to antigenic stimulus, inflammatory cell apoptotic process, wound healing involved in inflammatory response, and leukocyte chemotaxis involved in inflammatory response. KEGG results showed enrichment for multiple KEGG terms associated with inflammation, including autoimmune thyroid disease, graft-versus-host disease, herpes simplex infection, influenza A, and natural killer cell mediated cytotoxicity.

### 3.3. lncRNA Target Gene Prediction and Target Gene GO and KEGG Analysis

**Figure 4:**
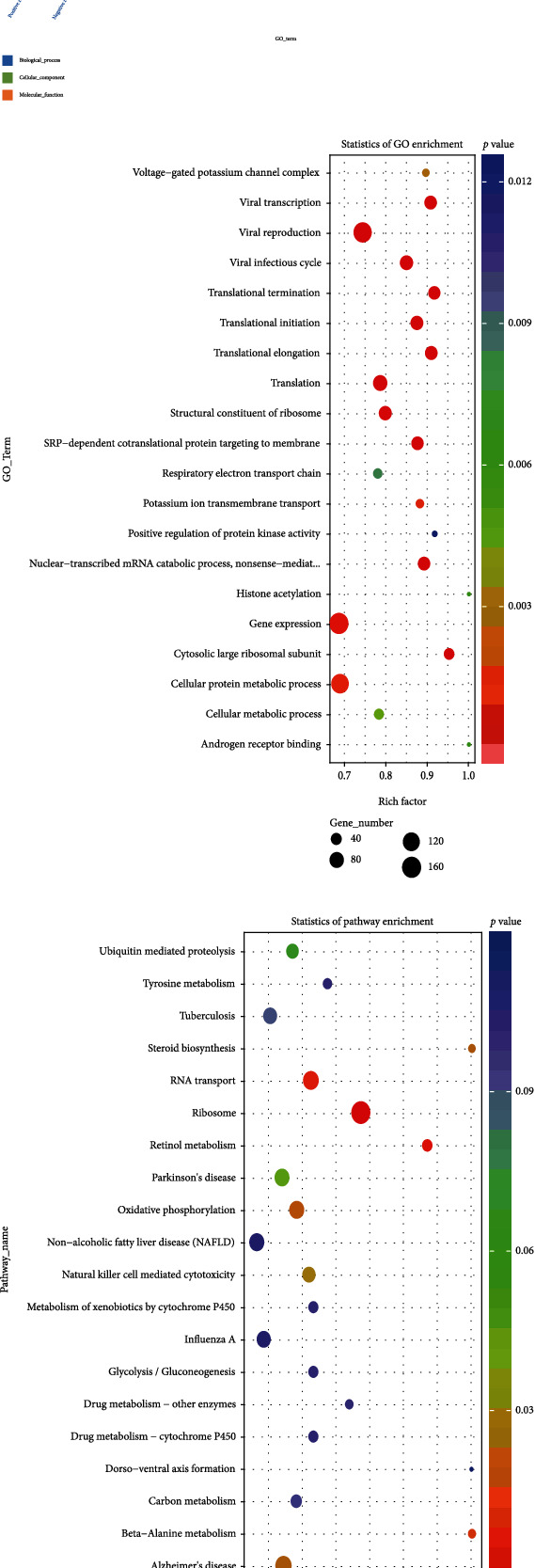
(a) GO enrichment histogram, (b) GO enrichment histogram, and (c) KEGG enrichment scatter plot show the functional profiles of differentially expressed lncRNAs targeting gene parental genes in healthy individuals and diabetic patients.

We then analyzed GO and KEGG analyses of lncRNA-targeted gene mRNAs, as shown in Figure 4. lncRNA regulation is divided into two main categories: cisregulation and transregulation. Cisregulation means that lncRNAs regulate the expression of adjacent genes. lncRNA cisregulation target genes are primarily predicted based on positional relationships, defining the existence of differentially expressed lncRNAs and mRNAs within 100 kbp upstream and downstream in chromosomes, and lncRNAs constitute cisregulation. We used cisprediction: a gene with a distance less than 100 kb from the lncRNA was selected as the target gene for cisaction.

### 3.4. Inflammation-Related lncRNA Screening and KEGG Analysis

**Figure 5:**
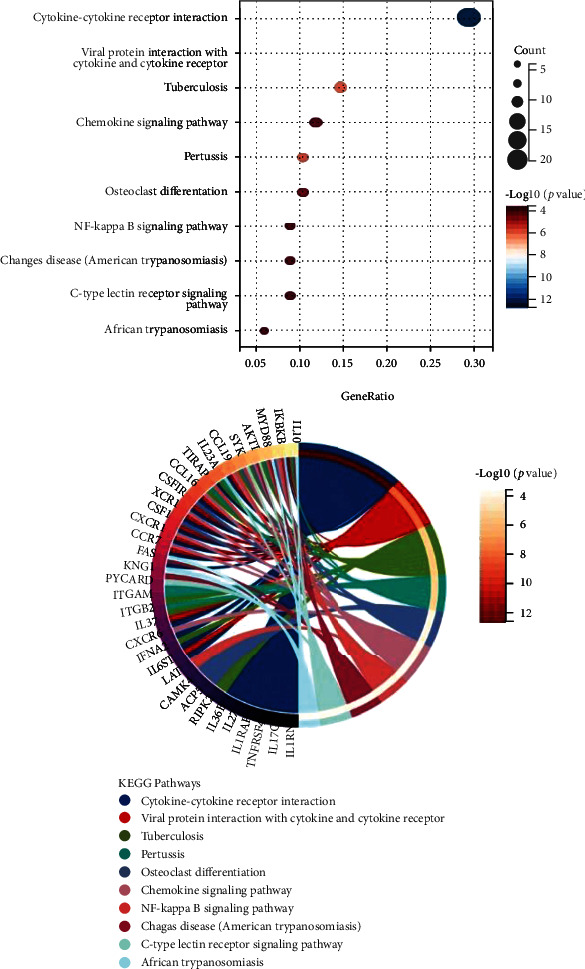
Key lncRNA target genes and KEGG pathway analysis results. The key lncRNA target genes are IL1RN, IL17C, TNFRSF4, IL1RAP, IL27, IL36B, RIPK2, ACP5, CAMK4, LATIL6, STIFNA2, CXCR6, IL37, ITGB2, ITGAM, PYCARD, KNG1, FAS, CCR7, CXCR1, CSF1, XCR1, CSF1R, CCL16, TIRAP, IL23A, CCL19, SYK, AKT1, MYD88, IKBKB, and IL10. The KEGG pathway analysis revealed cytokine–cytokine receptor interactions, viral protein-cytokine with cytokine and cytokine receptor, tuberculosis, chemokine signaling pathway, pertussis, osteoblast differentiation, NF-*κ*B signaling pathway, Chagas disease, C-type lectin receptor signaling pathway, and African trypanosomiasis.

Inflammation serves as a key component of diabetes. It is important to find mRNAs associated with inflammation. We identified inflammation-related terms in mRNA differential gene GO analysis, which included regulation of acute inflammatory response, chronic inflammatory response, negative regulation of inflammatory response to antigenic stimulus, inflammatory cell apoptotic process, wound healing involved in inflammatory response, and leukocyte chemotaxis involved in inflammatory response. Based on the results of differential analysis, 108 mRNAs were obtained by screening with corrected *p* values (*padj* ≤ 0.01) and differential fold change values > 2 − fold, i.e., |log2(*FC*)| > 1. Then, we used these 108 mRNAs for KEGG analysis, and the KEGG results (Figure 5) identified NF-*κ*B as one of the key inflammation-related pathways in diabetes. KEGG was used to analyze the pathways associated with inflammation in diabetes and could provide a basis for subsequent exploration of the mechanisms associated with inflammation in diabetes. To further find the lncRNAs associated with inflammation, we obtained lncRNAs associated with inflammation from 108 mRNAs by cis- and trans-lncRNA-mRNA pairs.

### 3.5. Results of lncRNA-mRNA Coexpression Network Graph Analysis

**Figure 6:**
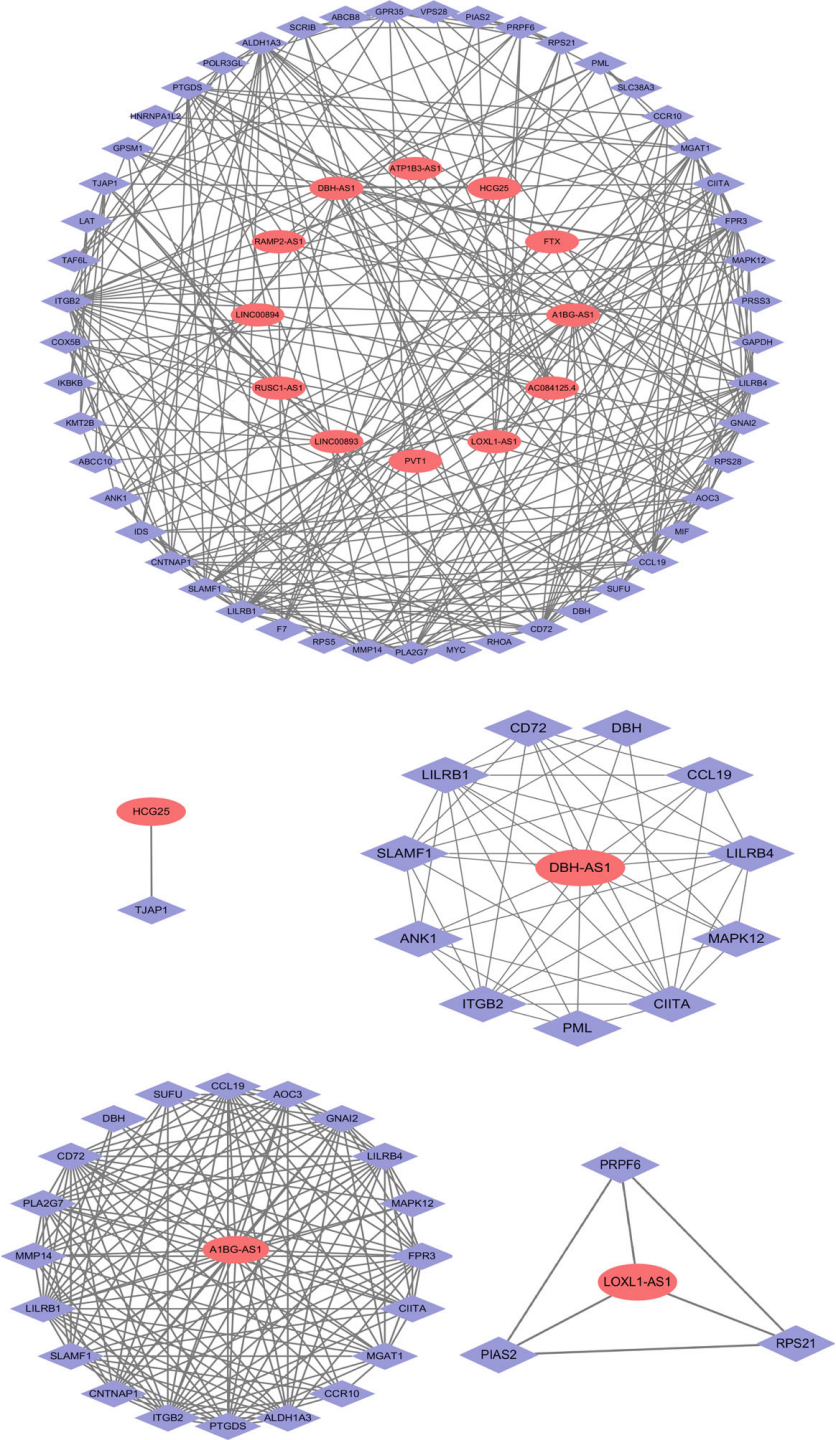
lncRNA-mRNA coexpression network. The purple dots represent mRNAs, and the red dots represent lncRNAs.

lncRNA-mRNA coexpression network (Figure 6) analysis using ncFANs v2.0 and lncRNA-mRNA coexpression network map using Cytoscape. Twelve candidate lncRNAs were screened according to the degree size and the results of differential analysis and subsequently validated by clinical samples. The top 12 dysregulated lncRNAs in type 2 diabetes mellitus are shown in Table 2.

### 3.6. RT-qPCR Experimental Validation

**Figure 7:**
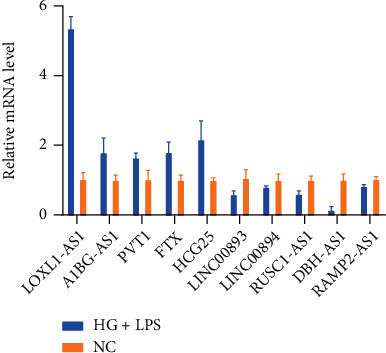
Relative expression levels of key lncRNAs in control THP-1 and HG+LPS-induced THP-1 cells as determined by RT-qPCR. The normal control (NC) with glucose (5.5 mmol/L) and HG+LPS group with glucose (25 mmol/L) + LPS (100 ng/mL) + IFN-*γ* (20 ng/mL) in RPMI 1640 culture medium were used to culture THP-1-induced macrophages for 2 days.

We performed RT-qPCR validation for ten candidate genes. These genes included LOXL1-AS1, A1BG-AS1, FTX, LINC00893, LINC00894, HCG25, RUSC1-AS1, DBH-AS1, RAMP2-AS1, and PVT1. Two of these genes (AC084125.4 and ATP1B3-AS1) have no sequence currently available on NCBI due to their novel nature. As shown in Figure 7, the RT-qPCR results confirmed that five genes, including LOXL1-AS1, A1BG-AS1, FTX, PVT1, and HCG25, were upregulated in the HG+LPS-induced THP-1 cell model, and five genes including LINC00893, LINC00894, RUSC1-AS1, DBH-AS1, and RAMP2-AS1 genes were downregulated in the HG+LPS-induced THP-1 cell model.

## 4. Discussion

Type 2 diabetes mellitus (T2DM) is a classic metabolic inflammatory disease that is regulated by a combination of environmental and genetic factors [10]. Inflammation-associated epigenetics is the central pathological mechanism leading to beta-cell dysfunction and insulin resistance [11]. With the development of molecular biotechnology, ncRNAs, which were once considered “noise,” play a critical role in various biological processes. lncRNAs are transcripts that are greater than 200 nucleotides in length [12]. By interacting with DNA, RNA, or proteins at the transcriptional, translational, and posttranslational levels, lncRNAs are involved in many cellular processes [4, 13]. lncRNA microarrays quickly and efficiently screen for differential lncRNAs associated with diseases or specific phenotypes to provide a theoretical basis for the occurrence, development, and prevention of human diseases. The common current approach to lncRNA functional studies is to infer the function of lncRNAs from functionally known target genes [14, 15]. A lncRNA-miRNA-mRNA network may be constructed from the prediction results of various prediction tools to link lncRNAs with target genes [16]. It is also possible to construct lncRNA-mRNA coexpression networks [17, 18] that predict the target genes of lncRNAs. The candidate lncRNAs were validated using RT-qPCR or Northern blotting to determine their expression differences.

The present study used a bioinformatic approach to construct a lncRNA-mRNA coexpression network map of peripheral blood mononuclear macrophages in T2DM. Based on the results of differential analysis, lncRNAs with a corrected *p* value (*padj* ≤ 0.01) and differential fold change value > 2 − fold, i.e., |log2(*FC*)| > 1, were screened, and lncRNAs from the commonly used noncoding RNA databases Ensembl and GENCODE were selected. Based on the above conditions, 769 differentially expressed lncRNAs and 3847 differentially expressed mRNAs were identified. A total of 108 mRNAs associated with inflammation were obtained from GO enrichment analysis based on the results of differential expression analysis. lncRNA-mRNA pairs were formed using cismethods to find differential lncRNAs. lncRNA-mRNA coexpression network analysis was performed using ncFANs v2.0 and drawn using Cytoscape. lncRNA-mRNA coexpression network maps were obtained for A1BG-AS1, AC084125.4, RAMP2-AS1, FTX, DBH-AS1, LOXL1-AS1, LINC00893, LINC00894, PVT1, HCG25, RUSC1-AS1, and RUSC1-AS1. Some of these lncRNAs, including FTX, PVT1, LINC00893, and LINC00894, are both up- and downregulated. It is because of different probes used for different gene transcripts. The presence of different transcripts for a gene may result in both up-and downregulation of gene expression due to alternative splicing, alternative promoter usage, alternative initiation, and ribosomal frameshifting [19].

We finally obtained ten key genes with stable RT-qPCR results as shown in Figure 7. Two genes were not shown because they were so new that NCBI did not have a primer design for the gene sequences. Among the key lncRNAs we identified, FTX, PVT1, and LOXL1-AS1 are currently more studied. FTX is involved in medullary thyroid cancer, osteosarcoma, pancreatic cancer, and other diseases [20–22] and plays an important role in the development of inflammation. Research shows that FTX improves the inflammatory response of microglia by competitively binding to miR-382-5p [23]. Macrophage migration inhibitory factor (MIF) genes associated with FTX in the coexpression network map are closely associated with diabetic inflammation [24–26]. Therefore, it is possible that FTX is involved in the inflammatory response in diabetes through MIF. Interestingly, RT-qPCR revealed that FTX was upregulated in osteosarcoma [27] and was reduced in myocardial I/R injury patients' serum and H/R-stimulated H9c2 cells [28]. This may be because the expression of FTX is inconsistent in different tissues. In our study, FTX was upregulated. Therefore, we hypothesize that FTX is a risk factor for diabetic inflammation. PVT1, one of the more studied lncRNAs, is closely associated with diabetes. The downregulated PVT1 is involved in gestational diabetes and preeclampsia via the regulation of human trophoblast cells [29]. It has been reported that PVT1 could accelerate the progression of diabetic nephropathy by promoting extracellular matrix accumulation and increasing the expression of fibronectin 1 [30]. This is consistent with our RT-qPCR results. LOXL1-AS1 is involved in liver cancer [31], gastric cancer [32] thymoma, and thymic carcinoma [33] by competitively binding miRNAs. There are currently no studies on LOXL1-AS1 in diabetes inflammation. However, LOXL1-AS1 is involved in the inflammatory response in coronary epicardial adipose tissue and osteogenic joints through a miRNA competitive binding mechanism [34, 35]. This provides evidence for subsequent studies of diabetic inflammation.

HCG25, A1BG-AS1, DBH-AS1, RUSC1-AS137, RAMP2-AS1, LINC00893, and LINC00894 are genes that are currently being studied mainly in cancer. No research has been reported in the direction of diabetes. Research has shown that HCG25 is upregulated in liver cancer [36] and A1BG-AS1 to be highly expressed in breast cancer tissues and cell lines. This is consistent with the results of our study. LINC00893 [37], LINC00894 [38], and RAMP2-AS1 [39] are protective factors for colon, thyroid, and breast cancer, respectively. Interestingly, RAMP2-AS1, LINC00893, and LINC00894 are also downregulated in our study model. LINC00839 and RAMP2-AS1 were identified as key RNAs in childhood obesity [40]. Obesity is believed to be a promoter of type 2 diabetes mellitus. This also provides the basis for the study of diabetes. However, DBH-AS1 and RUSC1-AS1 were inconsistent with our findings. DBH-AS1 expression was upregulated in HCC tissues and cells [41]. RUSC1-AS1 is reported to be upregulated and acts as an oncogene in hepatocellular carcinoma, cervical cancer, and breast cancer [42]. This may be due to the fact that gene expression is inconsistent in different tissues and in different models. Meanwhile, the mechanism of action of core genes in different tissues of diabetes still needs further in-depth study. Cancer is also an inflammatory disease, and cancer and diabetes share the same inflammatory pathogenesis, so we speculate that these genes also play an important role in diabetic inflammation. AC084125.4 and ATP1B3-AS1, as relatively new lncRNAs, have not been reported in the literature. This could be a new research direction in the future.

Most of our predicted KEGG pathways for key lncRNAs were associated with inflammation. Previous studies have shown that the NF-*κ*B signaling pathway is one of the most important signaling pathways in inflammation. Our KEGG pathway analysis also showed NF-*κ*B to be a relevant pathway for diabetes inflammation. In summary, we also show the validity of the network constructed in this study, and the key lncRNAs obtained are representative, which also fully indicates that this lncRNA-mRNA coexpression network map plays a potential role in the development of inflammation in T2DM. These genes are rarely reported in the literature, and fewer studies have been performed in diabetes-related fields. These genes may be the focus and direction of future research.

## 5. Conclusion

There are differentially expressed lncRNAs and mRNAs between patients with diabetes and healthy people. lncRNAs and mRNAs are extensively associated with each other and form coexpression networks. Differentially expressed lncRNAs play an important role in diabetic inflammation and influence the development of type 2 diabetes by regulating the corresponding mRNAs. The ten key genes we obtained, LOXL1-AS1, A1BG-AS1, FTX, LINC00893, LINC00894, HCG25, RUSC1-AS1, DBH-AS1, RAMP2-AS1, and PVT1, may become potential therapeutic targets for the future treatment of inflammation in type 2 diabetes, and these findings will help us better understand the potential mechanisms of lncRNAs in the prevention of diabetic inflammation. These results provide new perspectives and guidance for future exploration of functional changes in genes and signaling pathways associated with type 2 diabetes.

## Figures and Tables

**Table 1:** The sequences of PCR primers.

Gene name	Primer sequence (forward)	Primer sequence (reverse)
A1BG-AS1	CTCCCTCTGTTTGGTCCCAT	CCACCAGTCCTTGTCTGAGT
RUSC1-AS1	CTCCCTCTGTTTGGTCCCAT	CCACCAGTCCTTGTCTGAGT
RAMP2-AS1	CCACCACCACACACACAAAT	AGACCAGCAGGTGTGAATGA
LINC00893	CGGGAAATTGCTGGAGAAGG	TCAGGAGGCTCATGGAACAG
LINC00894	CCCTGGAGAAGCTTGAGGAA	ACTTGCACTTCCTCTCGTCA
DBH-AS1	GTGGCTGGACCTCACTGTAT	GTGGCTGGACCTCACTGTAT
LOXL1-AS1	ATCTGCCTCTCCTGTGTAGC	ATCTGCCTCTCCTGTGTAGC
HCG25	CGGGCTAATAGCAGGAGGAA	CGGGCTAATAGCAGGAGGAA
PVT1	CTTGGAGGCTGAGGAGTTCA	CTTCAGGCCTCTTTGACAGC
FTX	GCCCAGCAAGTTCATCAGAG	CTGCATGGTCACTCACATGG
GAPDH	TCTGGCACCACACCTTCTA	TCTGGCACCACACCTTCTA

**Table 2:** The top 12 dysregulated lncRNAs in type 2 diabetes mellitus.

lncRNA	Sequence	Regulation	log2FoldChange	*p* value	Chr	Target mRNA
A1BG-AS1	ATATTGCTGAGCACCTGCTGCATGCTCTCTGTCCTGCTCAGAGCCAGTACCTGGATGGAG	Up	3.87	<0.05	Chr19	CCR10, MGAT1, CIITA, FPR3, MAPK12, LILRB4, GNAI2, AOC3, CCL19, SUFU, DBH, CD72, PLA2G7, MMP14, LILRB1, SLAMF1, CNTNAP1, ITGB2, PTGDS, ALDH1A3
AC084125.4	TTCTCTGTCCAGTTCCGGGAAAGCACTGGCCGTCCTGCCTGCCTGTGGCTGTTCCTGCTA	Up	3.14	<0.05	Chr8	TAF6L, POLR3GL, SCRIB, ABCB8, GPR35, VPS28
RAMP2-AS1	GTAAAAAGTGCTAGGGCCTTCTACACCTTGAAATTATATGTGGAGCATCCTGCAATAAGC	Down	-1.51	<0.05	Chr17	SLC38A3, GAPDH, F7, PRPF6
FTX	AAGGAACAGGTATCTCAGATTCCCAGTTCTCAATCTTCCTTCCTCCTTACAAGATACAAG	Up	2.23	<0.05	chrX	MIF, GAPDH, IDS, HNRNPA1L2
	AGGGTCCTCTCAGTGATTAAGGATTATGAAGTATTAATTCTTCCACTGATATCTCAAAAG	Down	-1.9			
DBH-AS1	ATCAGCCACTGCTGTGATATACTGAGCACCTCCTCTAGAGAGAATAAAGGACTGACAAAG	Down	-1.10	<0.05	Chr9	PML, CIITA, MAPK12, LILRB4, CCL19, DBH, CD72, LILRB1, SLAMF1, ANK1, ITGB2
LOXL1-AS1	TTCCAAGGAGAAGCTCTGGAAAGTTCTGCCTGTCTCAGCACCTGGCTGGATCGGAAACGA	UP	2.88	<0.05	Chr15	PIAS2, RPS21, PRPF6
LINC00893	AAGTCAGTATTCACTTAAGCACTCCAAATTACCCAGCACACCCTGCCTGCATGGCGCTGC	Up	1.47	<0.05	chrX	RHOA, ABCC10
	GTGCAAAGTATGTCAGAAAGCAGATGGAAGCCAGGCCCCCTCCTGAAAGAGGCTCCTTGA	Down	-1.22			
LINC00894	TGGGTCATCCAGGAAACATACTCACCACTGTCTGTTCTCTGAGTTTTCATTTCCAGGCAT	Up	3.3	<0.05	chrX	ABCC10, KMT2B, IKBKB, TJAP1, LAT
	GCAGCAGGTGAGTTTCCTCACTTGGGTTTCACTGTCAGCATTTTAAAAAGGCAAACTGGT	Down	-1.34			
PVT1	AACTATTAAGGGGAAACAAAAGTGTTCTTAGGAGTCCTGCTGTCACTGTGGATTGAGCCG	Up	2.12	<0.05	Chr8	MYC, SCRIB, KMT2B, IKBKB, LAT, TJAP1
	AGGGTTGAGATCTCTGTTTACTTAGATCTCTGCCAACTTCCTTTGGGTCTCCCTATGGAA	Down	-1.75			
RUSC1-AS1	ATTTTCAAGTGTCTCTACGTAGCTAAAATCCCAAGCTTCCCTTCCCTATCCCAAATATTG	Down	-1.47	<0.05	Chr1	TJAP1, LAT
HCG25	GGTATAGCCATCCTTATCATCAAACCCTCTTTTCTGGTATTCTCTCAATCCAGTCTTTCA	Up	1.92	<0.05	Chr6	TJAP1
ATP1B3-AS1	AAATGCATTTAAAACCTTAAGGGGACCCAGCACTCCATCCAAGCTTATCTATGCAGCCTG	Up	2.24	<0.05	Chr3	LAT

## Data Availability

The datasets generated during and/or analyzed during the current study are available from the corresponding author on reasonable request.

## Abbreviations

DM: Diabetes mellitus
T2DM: Type 2 diabetes mellitus
lncRNA: Long-stranded noncoding RNA
miRNAs: MicroRNAs
ceRNAs: Competitive endogenous RNAs
MIF: Macrophage migration inhibitory factor
CIITA: MHC II transactivator.
